# The Corporate Purpose of Spanish Listed Companies: Neurocommunication Research Applied to Organizational Intangibles

**DOI:** 10.3389/fpsyg.2020.02108

**Published:** 2020-10-06

**Authors:** Luis Mañas-Viniegra, Igor-Alejandro González-Villa, Carmen Llorente-Barroso

**Affiliations:** Department of Applied Communication Studies, Complutense University of Madrid, Madrid, Spain

**Keywords:** corporate purpose, brand purpose, purpose-driven companies, IBEX 35, intangible economy, neuromarketing, eye tracking, galvanic skin response

## Abstract

Purpose driven companies have developed their corporate culture with a commitment to stakeholders, Sustainable Development Goals, and social responsibility, prioritizing the management of organizational intangibles over capital. The overall objective of this research is to gain knowledge regarding the attention and emotional intensity registered by young Spanish university students when visualizing corporate purpose versus corporate visual identity, as well as the image of the Chairman of the main Spanish companies quoted on the IBEX 35. The techniques of eye tracking and galvanic skin response have been used with 31 Spanish university students. The results suggest that brands with the highest brand equity in the Interbrand (2019) ranking are also the ones that receive the highest levels of attention and emotional arousal, and that a well-formulated corporate purpose is not enough to satisfy the public if company credibility is low due to previous perceptions of an organization.

## Introduction

Purpose-driven companies are those that have redefined their corporate culture in order to introduce and enhance corporate purpose or brand purpose as an evolution of theories such as shared value (Porter and Kramer, 2011), or triple bottom line (Elkington, 1998). Their aim is to provide public benefits consistent with Sustainable Development Goals, which in turn helps to improve the organization’s performance and contribution to solving or alleviating some of the social or environmental problems of the society in which they conduct their business (Fischer et al., 2019).

In recent decades, there has been a surge in corporate social responsibility and sustainability that has prompted a comprehensive business transformation (Quinn and Thakor, 2018) in which corporate purpose has managed to merge a commitment specifically stated by companies with a greater objective that society desires and values (Clarke, 2015; Wilson, 2017; Berghoff and Kelley, 2019). As a result, important changes have taken place in large organizations. One example is the shift from the debate over whether stakeholders should be exclusively people (da-Silva et al., 2019) to the reality that companies consider the environment to be a priority stakeholder (Mañas-Viniegra and González-Villa, 2019). However, this approach, which is both responsible and sustainable, can lead to friction with shareholders, who often put their own economic interests first (Besley and Ghatak, 2017).

Despite the fact that enterprises have always combined their commercial activities with a public purpose, the ‘Friedman doctrine’ (Friedman, 1970) of maximizing profit started a shift in thinking that has led to the current radical redefinition of the corporate mission through corporate purpose. The objective is to promote greater social well-being rather than maximize shareholder value (Mayer, 2018), thereby providing the basis for improving corporate social responsibility (Grinbaum and Groves, 2013).

However, for organizations it has always been relevant to formally define their corporate culture or organizational culture, which is composed of a set of values, beliefs and standards that are shared and adopted as their own by the members of an organization, which are transmitted to the new members who enter the company (Peters and Watterman, 1982; Schein, 1990). Corporate culture conditions the individual and group behavior of its members by establishing rules of conduct (O’Reilly et al., 1991; Shin et al., 2012). Its definition and internalization produce positive or negative behavior in an overall, general way within the organization (Wiewiora et al., 2013). Corporate culture is essentially established by the mission, vision and corporate values of the organization.

The mission is the reason for being, or the purpose of an organization (Drucker, 1973), and it states the way in which the entity intends to fulfill this mission through a long-term vision of the future (Johnson and Scholes, 2001). Using the mission and vision as a starting point, corporate values are established that shape the personality of the corporate brand in a relatively stable manner (Christensen et al., 1982), and guide the behavior and attitudes of the members of the organization. This also benefits the relationship with stakeholders (Freeman, 1984). As a result, values have traditionally been considered the main element of corporate culture (Deal and Kennedy, 1982). In fact, once values have been established, a small proposal for change ends up generating resistance from the organization’s members who have already assimilated these values to a great extent (Ogbonna and Harris, 2014).

With the advent of corporate purpose, there has been a necessary evolution of corporate culture of organizations toward the inclusion of universal values of a higher order, which are socially accepted, and which in turn awaken the emotions of stakeholders by satisfying their desire for solidarity, freedom, happiness and respect when it comes to achieving success. This leads to the development of corporate culture that is committed to society, and consequently enhances the credibility and reputation of organizations (Van-Lee et al., 2005), based on the confluence of business excellence, ethical behavior, and corporate social responsibility as values shared by all of those who belong to an enterprise (Drucker, 1981; Kotler and Heskett, 1992; Solomon, 1992; Chell et al., 2016).

Thus, corporations prioritize intangibles over capital (Haskel and Westlake, 2017), and have the potential not only to determine their purpose, but also to ensure that the premise of ‘doing well by doing good’ is fulfilled (Mayer, 2018). The goal that states, ‘avoid doing harm’, is thereby exceeded in order to aspire to the objective that states, ‘do good’ (Scherer and Voegtlin, 2018).

Corporate purpose has been identified as one of the fundamental elements in improving corporate culture among banks in the United Kingdom (Cox and Soobiah, 2018). Moreover, in France, the United Kingdom and the United States, it has already been written into law that a corporation must be managed while bearing in mind the social and environmental impact produced by its activity, and to do so, the entity must define its social or environmental purposes in its by-laws (Segrestin et al., 2020). Even though the usefulness of formally incorporating purpose into the constituent documents of corporations has been demanded (Lin, 2019), there is a risk of reducing the capability that an organization needs in order to adapt to the evolution of society, which would lessen the control it could exercise due to having a purpose that has already been defined (Levillain and Segrestin, 2019).

In short, despite the growing importance of corporate purpose, there is no clear or consistent definition beyond that of its long-term orientation and commitment to stakeholders (Waitzer and Sarro, 2018). Therefore, this purpose has been defined more clearly in the professional area rather than the academic field, which still has a scant amount of literature: ‘Businesses need to encapsulate it into a clear purpose. That purpose should be compatible with Sustainable Development Goals, and it needs to shape the way the business is both designed and run’ (Deloitte, 2017, p.3).

Organizations are starting to become aware of their purpose, thereby clarifying their reason for being in order to fulfill their responsibilities related to economics, social issues, governance, ethics, and environmental matters, which they clearly express (White et al., 2017). Companies committed to fulfilling their responsibilities also disclose their best practice in Corporate Social Responsibility to stakeholders (Balmer and Greyser, 2006), based on the benefits resulting from the establishment of an ethical corporate identity (Hildebrand et al., 2011). This issue promotes the incorporation of best practice communication in the brand’s core value proposition, thereby strengthening brand equity (Muñiz et al., 2019).

Although it must be consistent and credible, merely stating corporate purpose does not guarantee that it will contribute positively to society (Serafeim, 2018), or that the impact on company profits will be positive (Quinn and Thakor, 2018). In spite of this, a correlation between purpose and the strengthening of corporate reputation has been confirmed (Henderson and Van-den-Steen, 2015), and it is not mere coincidence that the 50 fastest growing brands worldwide in 2018 have defined their corporate purpose clearly, and have increased their shareholder value tenfold (Cardona and Tolsá, 2018).

It has traditionally been considered that the values of an organization should be compatible with the management style of its leaders (Blanchard et al., 1997), and next in order of importance, with the values of its employees (Vveinhardt and Gulbovaite, 2017). However, some research that has focused on the influence of corporate purpose on financial performance suggests that it is not senior management, but rather the middle ranks of the organization, who drive this correlation (Gartenberg et al., 2019), which differs from classical theories that assign responsibility for fostering a common sense of purpose to senior management (Bartlett and Goshal, 1993). In spite of this, the commitment and motivation of the organization’s leaders is necessary in order for this corporate change to take place (Seah et al., 2014), and subsequently for the entity to be evaluated by initiatives that are starting to emerge, such as the B-Corp certification (Muñoz et al., 2018).

Listed companies have been the pioneers in defining corporate purpose as they have also been the first to disassociate their product brands from their corporate brand (Olins, 2009), having enhanced the intangibles within their organizations through values, reputation, transparency, social responsibility and corporate governance (Mañas-Viniegra, 2017). Even though only 37% of listed Spanish companies and 28% of listed Portuguese companies have specifically defined their corporate purpose, the analysis of these enterprises has made it possible to identify the basis upon which their purpose is outlined.

“The first pillar is built on concepts such as the future, the environment, the world, sustainability, social responsibility and life. These corporate purposes convey the intention of organizations to promote strong global improvement as a result of their activity. The second pillar revolves around the public that will benefit from this overall improvement: citizens, people, society in general, customers and shareholders, or other sectors of the public directly related to the business” (Mañas-Viniegra et al., 2020c, p.21).

As a continuation of previous research, and faced with a future that will include the commencement, development, and spread of corporate purpose in all companies during the next decade, this research uses neuromarketing techniques to analyze the cognitive perceptions of young Spanish university students, who represent a new generation more concerned with social and environmental issues (Gifford and Nilsson, 2014; Bhattacharya, 2019). This research seeks to provide answers to the following research questions:

(RQ1) Does corporate purpose awaken an emotional response in an audience of young university students? (RQ2) Does corporate purpose attract more attention than corporate visual identity, or the image of the President who leads the organization? (RQ3) Are there differences between the attention and emotion directed at brand purpose?

## Materials and Methods

The overall objective of this research is to understand the attention and emotional intensity experienced by young Spanish university students when visualizing images of corporate purpose vs. corporate visual identity, and the image of the Chairman of the main Spanish companies quoted on the IBEX 35.

The sample of the experiment is composed of six companies among the top ten in the ranking of Best Spanish Brands (Interbrand, 2019), which currently have a defined purpose: Zara, Movistar, Santander Bank, BBVA, CaixaBank and Iberdrola. This sample is not representative of diverse business sectors, but rather of the most highly valued companies, which means that some sectors are not represented, yet others that are more powerful have significant representation.

To this end, the following specific objectives have been established:

1. O1: To identify the attention and emotional intensity that the subjects display toward corporate purpose.
2. O2: To establish the differences in attention and emotion between the diverse elements visualized in the stimuli: corporate visual identity (logo), image of the Chairman, and corporate purpose.
3. O3: To analyze differences in attention and emotion based on the presence or absence of corporate purpose.
4. O4: To identify possible differences that may occur depending on the gender of the subjects.

This research uses two non-intrusive techniques of neuromarketing, or applied neurocommunication (Cuesta-Cambra et al., 2017). These have been fully validated (Morin, 2011) for recording the cognitive processing of subjects based on Neuroscience, Psychology and Economics (Madan, 2010). Moreover, they have a reliability rating for predicting the effectiveness of communication stimuli between 70% and 80% (Varan et al., 2015): eye tracking was used to measure the subjects’ attention to the areas of interest (AOI) of the stimuli presented, and galvanic skin response (GSR) was used to record the emotional intensity experienced by the participants.

On the one hand, eye-tracking records the subjects’ attention to the areas of interest (AOI) of the stimuli displayed. This is a biometric technique that differentiates between the attention focused on AOIs and the viewing of transitory areas where attention is not fixed (Duchowski, 2013). From the attention registered, the software generates heat maps in which you can visually see where the attention is concentrated with different intensities, depending on the color. Red represents the center of the heat map where attention is most intense. On the other hand, galvanic skin response (GSR), or electrodermal activity (EDA), identifies the emotional arousal from the small changes in the electrical conductance of the skin that are produced by sympathetic neuronal activity from phasic changes (Critchley, 2002).

Therefore, these two neuromarketing techniques allow us to record the unconscious responses of the participants to the visualized stimuli in the form of attention and emotional intensity, thereby allowing for the partial identification of whether or not an influence is produced, which is carried out by analyzing the cognitive and emotional processing (Bornstein and D’Agostino, 1992; Pieters et al., 2002; Goodrich, 2011). This overcomes the difficulty demonstrated by subjects in reporting their own perceptions, attitudes or behaviors in surveys or focus groups (Ariely and Berns, 2010). Two groups of 31 subjects each with gender parity participated in this research. They were randomly and voluntarily selected to form a convenience sample comprised of university students in the final year of their degree (on the verge of entering the labor market) in Madrid, the capital of Spain, which has a concentration of students from all regions of the country. Although the research fieldwork had to be completed earlier than planned in February 2020 due to the Covid-19 health crisis, the final sample size is adequate for a neuromarketing study, which is usually conducted with a sample of 15 to 50 subjects (Kerr-Gaffney et al., 2018).

The participants viewed six random stimuli interspersed with other stimuli related to brands, companies, or the job market, among others, without specifying which stimuli were of interest to the researchers. The first group (Group 1-G1) viewed only the image of the Chairman and the company logo (Table 1), while the second group (Group 2-G2) also viewed the branding purpose (Table 2).

**TABLE 1:** Areas of Interest (AOI) of the stimuli (Group 1-G1).

(a) Stimulus 1 (S1): Logo (AOI 1), President (AOI 2), All areas (AOI 3).	(b) Stimulus 2 (S2): Logo (AOI 1), President (AOI 2), All areas (AOI 3).	(c) Stimulus 3 (S3): Logo (AOI 1), President (AOI 2), All areas (AOI 3).
(e) Stimulus 4 (S4): Logo (AOI 1), President (AOI 2), All areas (AOI 3).	(f) Stimulus 5 (S5): Logo (AOI 1), President (AOI 2), All areas (AOI 3).	(g) Stimulus 6 (S6): Logo (AOI 1), President (AOI 2), All areas (AOI 3).

**TABLE 2:** Areas of Interest (AOI) of the stimuli (Group 2-G2).

(a) Stimulus 7 (S7): Logo (AOI 1), President (AOI 2), All areas (AOI 3), Purpose (AOI 4).	(b) Stimulus 8 (S8): Logo (AOI 1), President (AOI 2), All areas (AOI 3), Purpose (AOI 4).	(c) Stimulus 9 (S9): Logo (AOI 1), President (AOI 2), All areas (AOI 3), Purpose (AOI 4).
(e) Stimulus 10 (S10): Logo (AOI 1), President (AOI 2), All areas (AOI 3), Purpose (AOI 4).	(f) Stimulus 11 (S11): Logo (AOI 1), President (AOI 2), All areas (AOI 3), Purpose (AOI 4).	(g) Stimulus 12 (S12): Logo (AOI 1), President (AOI 2), All areas (AOI 3), Purpose (AOI 4).

The exposure to each stimulus was limited to 10 s, with a 3-s interval between stimuli to stabilize the registers of emotional intensity (Mañas-Viniegra et al., 2020a). However, each participant was given instructions in order to be able to move on to the next stimulus earlier by pressing a key. This limited the bias usually present in this type of study in which participants are asked to view stimuli, which implies that total attention is greater than it otherwise would be in an unobserved environment. The fact that the participants do not know which of the viewed stimuli are of interest to researchers allows for the differences between stimuli to be studied more realistically. By limiting the viewing to 10 s per stimulus, the software differentiates where attention is prioritized, since the young audience is more likely to quickly focus their attention on a stimulus with information that is relevant and of interest to them (Añaños-Carrasco, 2015). This is the way in which an exploratory study is defined with an intra-subject design and random assignment on the various levels of experimental treatment (image sequences).

This research has been reviewed and approved by the Research Ethics Committee of the Department of Applied Communication Studies of the Media and Communication Science School, Complutense University of Madrid.

For data collection, the Gazepoint GP3HD 150 Hz sampling rate eye tracking equipment was used along with a GSR Gazepoint Biometrics system, integrating the analysis of the recorded data in the Gazepoint Analysis UX Edition v.5.3.0 software. Statistical exploitation of the results was carried out using SPSS v.25 software.

All of the participants were notified of their voluntary participation and anonymous contribution, and informed consent was given following the guidelines of the Declaration of Helsinki. The dependent variables were the level of attention and peaks of emotional arousal recorded by the eye tracker and GSR systems, respectively. The independent variable was the gender of the participants, as the profile of the subjects was similar.

The quantitative analysis was carried out on the basis of the seconds that elapsed from the appearance of the stimulus to the first fixation, or Time From Fixation (TFF), the number of eye fixations, or Fixation Count (FC), the total number of seconds of attention toward each area of interest, or Total Fixation Duration (TFD), and the GSR peaks that appeared from each minimum-maximum pair starting from the beginning of the emotional activation. A qualitative content analysis was also performed using the attention toward the stimuli recorded by the heat maps, together with a conscious statement by the subjects about the positive, negative, or neutral emotion shown toward the AOI, which was carried out in a semi-mechanical, non-intrusive way using GSR Gazepoint Biometrics.

## Results

Group 1 was exposed to stimuli without corporate purpose, and this group recorded a higher total fixation duration (TFD) toward the two most valued brands in the Interbrand ranking (Table 3), Zara (TFD = 8.52), and Movistar (TFD = 7.35), far behind Iberdrola (TFD = 4.64). The brand with the worst total duration of attention was CaixaBank (TFD = 3.52). Similarly, Group 2 was exposed to the same stimuli, but with brand purpose included, and they also registered greater attention duration to Zara (TFD = 8.97) and Movistar (TFD = 9.06), but this time there was less difference in attention duration toward Iberdrola (TFD = 8.37). CaixaBank was again the brand with the lowest registered attention (TFD = 4.72). All of the stimuli that included corporate purpose obtained a longer total duration of attention in all areas of the stimulus (AOI 3) from Group 2 compared to Group 1, and the differences were statistically significant (*p* ≤ 0.001), except in the case of Zara, which recorded similar attention in both groups of subjects (*p* = 0.583).

**TABLE 3:** TFD (Mean).

AOI	S1	S7	*p*-Value	AOI	S2	S8	*p*-Value	AOI	S3	S9	*p*-Value	AOI	S4	S10	*p*-Value	AOI	S5	S11	*p*-Value	AOI	S6	S12	*p*-Value
AOI 1	4.55	2.79	*0.000	AOI 1	3.94	1.83	*0.000	AOI 1	2.19	1.69	*0.005	AOI 1	3.26	3.22	0.709	AOI 1	2.20	1.86	0.087	AOI 1	2.20	1.85	0.070
AOI 2	3.66	2.35	*0.000	AOI 2	3.56	2.15	*0.000	AOI 2	1.36	1.40	0.564	AOI 2	1.25	1.17	0.281	AOI 2	1.31	1.06	*0.001	AOI 2	2.44	2.30	0.983
AOI 3	8.52	8.97	0.583	AOI 3	7.35	9.06	*0.000	AOI 3	3.59	6.97	*0.000	AOI 3	4.55	6.89	*0.000	AOI 3	3.52	4.72	*0.000	AOI 3	4.64	8.37	*0.000
AOI 4	–	3.55	–	AOI 4	–	4.77	–	AOI 4	–	3.71	–	AOI 4	–	2.38	–	AOI 4	–	1.86	–	AOI 4	–	4.10	–

*U Mann–Whitney test between similar AOIs. ^∗^*p* < 0.05.*

The data on total duration of attention were confirmed by the total number of eye fixations (FC), also higher in all the stimuli with corporate purpose to which Group 2 was exposed (Table 4), with significant differences in all cases (*p* ≤ 0.001). Once again, Zara registered the highest number of eye fixations of all the stimuli presented, both in Group 1 (FC = 19.55) and in Group 2 with purpose (FC = 25.35), followed by Movistar (S2, FC = 18.58; S8, FC = 26.90) and Iberdrola (S6, FC = 12.65; S12, FC = 24.29).

**TABLE 4:** FC (Mean).

AOI	S1	S7	*p*-Value	AOI	S2	S8	*p*-value	AOI	S3	S9	*p*-Value	AOI	S4	S10	*p*-Value	AOI	S5	S11	*p*-Value	AOI	S6	S12	*p*-Value
AOI1	10.16	7.35	*0.000	AOI1	10.63	5.32	*0.000	AOI1	6.77	5.00	*0.001	AOI1	7.42	7.19	0.813	AOI1	6.71	5.40	*0.009	AOI1	6.55	5.94	0.248
AOI2	8.74	5.77	*0.000	AOI2	8.63	5.77	*0.001	AOI2	4.26	4.45	0.959	AOI2	3.32	3.45	0.954	AOI2	4.10	3.30	*0.007	AOI2	6.16	6.03	0.960
AOI3	19.55	25.35	*0.000	AOI3	18.58	26.90	*0.000	AOI3	10.97	21.77	*0.000	AOI3	10.71	17.68	*0.000	AOI3	10.58	13.48	*0.000	AOI3	12.65	24.29	*0.000
AOI4	–	12.10	–	AOI4	–	15.90	–	AOI4	–	12.48	–	AOI4	–	7.26	–	AOI4	–	5.27	–	AOI4	–	12.29	–

*U Mann–Whitney test between similar AOIs. ^∗^*p* < 0.05.*

Again, the worst register recorded was for CaixaBank, which obtained the lowest number of eye fixations for both stimuli (S5, FC = 10.58; S11, FC = 13.48).

The elapsed time from the appearance of each stimulus to the recording of the first visualization was immediate (TFF = 0.01), although analysis of this parameter will be relevant when analyzing the differences in attention between the areas of interest (AOI) highlighted in the stimuli (Table 5).

**TABLE 5:** TFF (Mean).

AOI	S1	S7	*p*-Value	AOI	S2	S8	*p*-Value	AOI	S3	S9	*p*-Value	AOI	S4	S10	*p*-Value	AOI	S5	S11	*p*-Value	AOI	S6	S12	*p*-Value
AOI 1	0.33	0.18	*0.035	AOI 1	0.25	1.09	*0.000	AOI 1	0.17	0.55	*0.000	AOI 1	0.11	0.11	0.993	AOI 1	0.12	0.16	0.623	AOI 1	0.20	0.14	0.370
AOI 2	2.38	1.44	*0.005	AOI 2	1.14	1.60	0.098	AOI 2	1.48	0.74	*0.003	AOI 2	2.44	2.41	0.688	AOI 2	1.49	1.56	0.834	AOI 2	1.19	2.10	0.109
AOI 3	0.04	0.01	0.415	AOI 3	0.01	0.01	0.710	AOI 3	0.01	0.01	0.459	AOI 3	0.01	0.01	0.432	AOI 3	0.01	0.01	0.357	AOI 3	0.03	0.01	0.083
AOI 4	–	5.68	–	AOI 4	–	0.70	–	AOI 4	–	2.18	–	AOI 4	–	3.68	–	AOI 4	–	2.40	–	AOI 4	–	2.56	–

U Mann–Whitney test between similar AOIs. *p < 0.05.

The brand logo registered greater attention duration than the image of the company President in all of the stimuli, except in the case of Iberdrola, which obtained greater attention duration in both stimuli (S6, TFD = 2.44; S12, TFD = 2.30), and with Telefónica, which was also higher in the stimulus with corporate purpose (S8, TFD = 2.15), due to the path taken after reading the purpose. The logo also registered first attention faster than the image of the company President in all cases, and a greater number of eye fixations, despite two exceptions without significant differences: those exceptions were Movistar in the stimulus with brand purpose (S8, FC = 5.77), and Iberdrola, also in the stimulus with purpose (S12, FC = 6.03).

**FIGURE 1:**
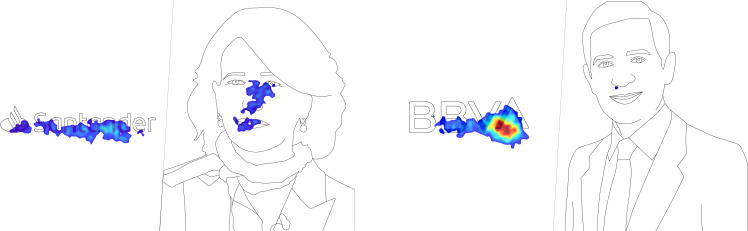
Heat maps of the stimuli.

It can be observed in the heat maps that the greatest attention toward the BBVA logo was focused on the last letter of the logo, due to the fact that it rises slightly, which coincides with the novelty of a recently renewed corporate visual identity (Figure 1).

**FIGURE 2:**
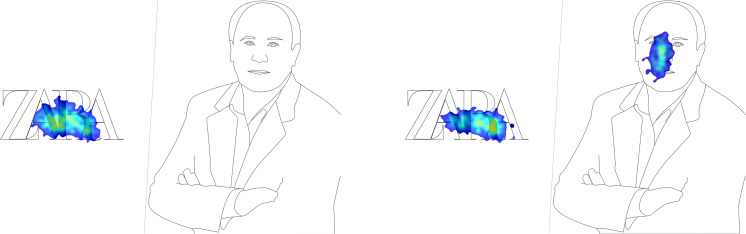
Heat maps of the stimuli.

Also noteworthy is the attention focused on Zara’s logo, described by the participants as “strange” due to the typography being too close together, a consequence of a recent redesign of their corporate visual identity (Figure 2).

**FIGURE 3:**
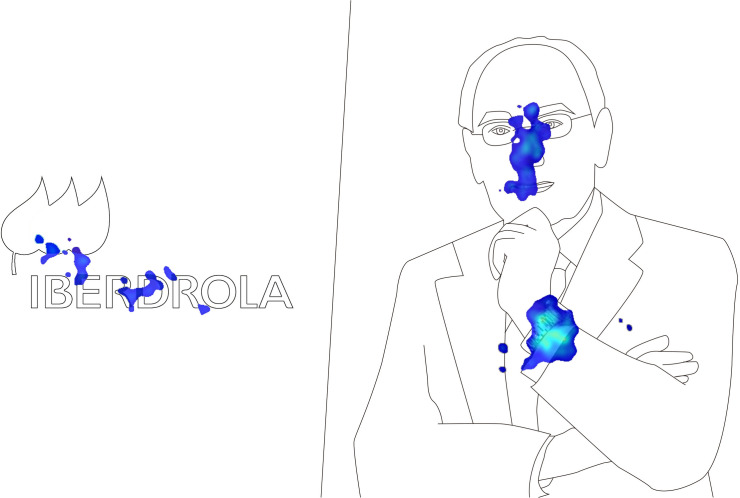
Heat maps of the stimuli.

Content analysis carried out based on the heat maps shows that the highest level of attention registered by the Chairman of Iberdrola was due to the attention focused on the watch shown in the foreground (Figure 3).

**FIGURE 4:**
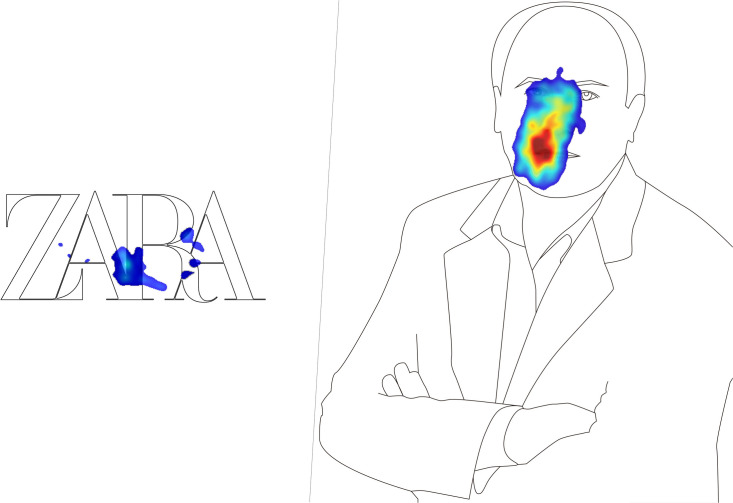
Heat maps of the stimuli.

The attention registered by Amancio Ortega’s face (Figure 4), on the other hand, was explained by the participants themselves in the informal conversations following the neuromarketing test, in which they said, “I wasn’t sure whether or not it was Amancio Ortega. He looks much younger in that photograph.”

**FIGURE 5:**
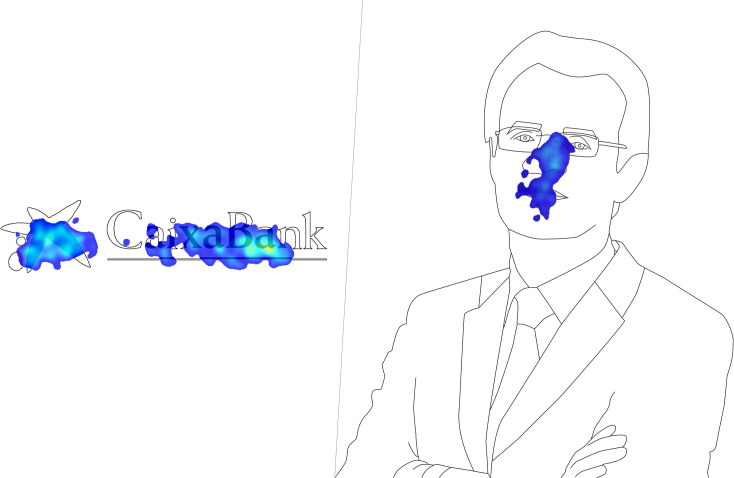
Heat maps of the stimuli.

The scant attention received by the Chairwoman of Banco Santander was explained by the participants, who simply said, “She didn’t look good in the picture.” They also mentioned their “lack of sympathy for banks”, an issue that can also be seen in the other two banks analyzed (BBVA and CaixaBank), which obtained the worst registers in all of the variables (Figure 5).

Zara and Movistar showed statistically significant differences (*p* ≤ 0.001) in attracting longer-lasting attention with more eye fixations on both the logo and the president in the stimulus without purpose, an issue that already anticipates that the purpose has attracted some attention that in Group 1 was directed toward other areas of interest. Group 2 visualized the stimuli with corporate purpose, and registered a longer total duration of attention toward the AOI of purpose in relation to the logo and the image of the company Chairman, except in the case of BBVA (S10, TFD = 2.38), where the duration of attention to the logo was higher (TFD = 3.22). Movistar’s corporate purpose registered the longest total length of attention (TFD = 4.77), followed by that of Iberdrola (TFD = 4.10), Santander (TFD = 3.71), Zara (TFD = 3.55), BBVA (TFD = 2.38) and La Caixa (TFD = 1.86). These data were supported by the number of ocular fixations, which showed an almost identical distribution. All corporate purpose stimuli were the last area of interest to be visualized, which is logical, as they were at the bottom of the stimulus, with the exception of Movistar, which was the first area of interest to capture the subjects’ attention (TFF = 0.70). The subjects explained this issue during the informal discussion after the neuromarketing test in which they stated, “This happened because we were reading the purpose, but we didn’t have time to finish reading it before the next stimulus appeared.”

**FIGURE 6:**
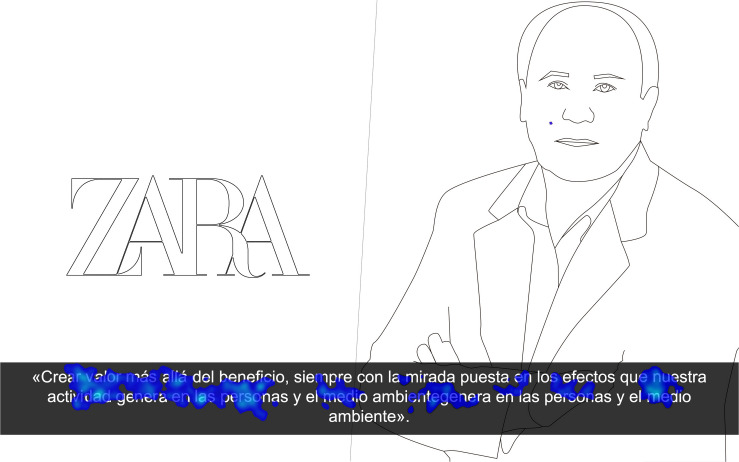
Heat maps of the stimuli.

It should also be noted that Zara’s corporate purpose had the largest number of words, and as the time allotted for each stimulus was limited to 10 s, it seems that the greater initial attention to the logo and the image of the President resulted in the attention toward purpose being diminished (Figure 6).

**FIGURE 7:**
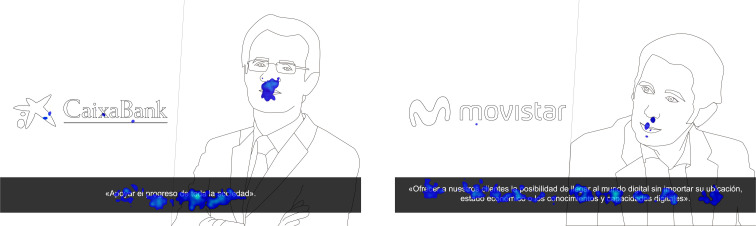
Heat maps of the stimuli.

Even though the longer length of Zara’s corporate purpose reduced the attention it received, La Caixa and BBVA, which presented their brand purposes with fewer words, were also the ones that got the least attention, so it seems that the length is not as relevant to the public as brand equity and a few easily recognizable visual components. As in the case of BBVA, the CaixaBank logo (in this case the logo icon) received a greater focus of attention than the other elements (Figure 7).

The gaze plots made it possible to check the visual journey made by the subjects while their attention was being registered. It was noted that after reading the corporate purpose, some subjects focused their attention on the President, and other participants focused on the company logo to a lesser extent. This behavior was mirrored in the registration of emotional arousal of the participants, which positively increased when a check was made once again of the brand that announced corporate purpose in the case of Zara and Movistar, but descended in the case of Iberdrola and the banks because of “the discredit of these companies, which include purpose only to improve their reputation”, as repeated by several participants in the semi-structured talks following the neuromarketing test.

There were no significant differences according to the gender of the participants, so this variable has been omitted in the analysis carried out.

The subjects’ conscious responses were positive toward Zara’s stimulus, neutral in the case of Telefónica, and neutral or negative in the case of the banks and Iberdrola.

**FIGURE 8:**
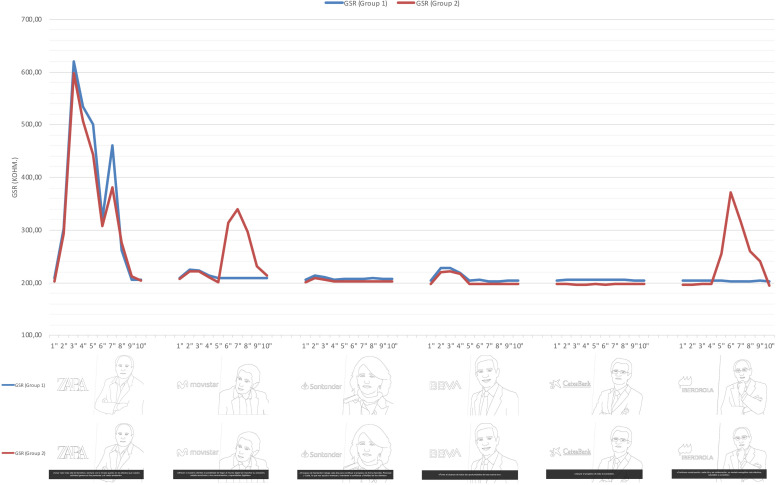
GSR peaks of the stimuli.

As for the emotional intensity based on the participants’ unconscious responses provided by the GSR data (Figure 8), it remained constant during the stimuli, with a moderate rise during the visualization of the logos.

There were two GSR peaks that occurred with the Zara logo and the image of its Chairman (620.72 kOhm. and 460.30 kOhm., respectively), as well as with the corporate purpose of Iberdrola and Telefónica (370.80 kOhm. and 340.22 kOhm., respectively).

## Discussion and Conclusion

Corporate purpose is currently undergoing professional and academic development, yet few listed companies in Spain have adopted this concept despite its increasing importance within the corporate culture of organizations, and the constant request from stakeholders for companies to focus on sustainable development objectives and corporate social responsibility.

The companies with the best rating on the Interbrand ranking in Spain are Zara and Movistar, which are the ones that registered the most attention toward their logo, the image of their President, and their corporate purpose. The emotional intensity of these two companies was also higher. However, the disaffection felt by young people toward banks resulted in CaixaBank, BBVA and Santander obtaining the worst results, both in attention and emotional arousal. One must consider that the level of affinity toward a brand is determined by the attraction and interest of the participants toward the brand (Aaker and Lee, 2001), and this influences the self-regulating objective of the subjects when they process the information (Petty and Cacioppo, 1979).

Even though Iberdrola came in last place in the Interbrand ranking of the companies analyzed, this brand was the second highest in registering a greater level of attention toward its purpose, despite the decrease in emotional intensity when the brand behind the purpose was visualized a second time. This result is consistent with corporate purpose identified in a successful case in previous investigations (Mañas-Viniegra et al., 2020b), even though the company in question does not seem to convey the credibility that such a purpose demands.

Even though word length does not appear to have influenced the results, participants noted that Zara’s purpose was excessively long and that the maximum stimulus display time ended before they could finish reading the text. In any case, the scientific literature has shown that young audiences easily focus their attention on areas that are interesting for them when time is limited (Añaños-Carrasco, 2015). Consequently, it can be concluded that in this case, corporate purpose was not the element they considered most important, but rather the visual aspects.

The inclusion of corporate purpose increased the total duration toward the overall content of all the stimuli, and toward purpose itself, which amassed the longest total duration of attention, partly due to the required reading time, even though there was no mandatory requirement to remain in the stimulus, and even less obligation to read the purpose.

These results are consistent with the fact that stakeholders are skeptical of the CSR communication of brands (Dhanesh and Nekmat, 2019), and of their communication of corporate purpose as well. Specifically, younger stakeholders express commitment to responsible organizations that are purpose-driven, but their actions do not yet reflect their attitudes (Dhanesh, 2020). Consequently, it may be in their interest to try to co-promote sustainable corporate brands with consumers (Lahtinen and Narvanen, 2020).

The logo registered more attention than the image of the President, unless there was some visual element that attracted attention, such as a watch in the foreground. Contributing to this situation were updates to corporate visual identity undertaken recently by some brands, such as BBVA and Zara. These results substantiate the greater attention and emotional arousal that the redesigned logos generate through the use of flat design (Gu and Yu, 2016; Mañas-Viniegra et al., 2020c).

Logos are complex stimuli comprised of various visual elements (Jiang et al., 2016), which reflect the identity of a brand, thereby influencing public attitudes (Kaur and Kaur, 2019). The design of a brand’s logo and its positioning have a significant impact on the perception of its products (Bettels and Wiedmann, 2019). The full meaning of a logo is achieved through the symbolic potential of its expressive forms (Llorente-Barroso and García-García, 2015), and is developed through connections generated by communication structures surrounding the brand (Kelly, 2017). The shape of a logo and its size are features that are powerful enough to influence the perception of a brand and its personality (Jiang et al., 2016; Cai and Mo, 2019), while its colors create a strong identification used to engender consumer confidence in the brand (Jin et al., 2019).

A logo change is seen as disruptive to the stability of a brand’s image (Villafañe, 1999; Stuart and Muzellec, 2004). Therefore, when faced with changes in corporate visual identity, it is advisable to guide consumers in their expectations, thus reducing the contrast between new logos and old ones (Grobert et al., 2016).

The main limitations of this research are the non-representativeness of the sample. Even though the size is adequate for neuromarketing studies, it is limited to Spanish university students.

The lack of funding for the research has resulted in the authors not having access to AFFDEX analysis of facial expression of emotions, which has been supplemented by registering emotional intensity together with a conscious statement of the type of emotion experienced by the participants.

It should also be noted that simulating a real environment in a neuromarketing laboratory is quite difficult (Mileti et al., 2016). However, this issue has the advantage of allowing for further insight into causation when combined with qualitative research (Berns et al., 2010).

Consequently, future lines of research should focus on carrying out cross-cultural investigations in order to establish socio-cultural distinctions among diverse geographical regions (Alsaleh et al., 2019), as well as the analysis of facial micro expressions when funding is obtained.

## Author’s Note

Due to copyright restrictions, the figures displayed in this article are vector graphics of those used as stimuli in the presented experiment, and as such do not correspond in the degree of definition and detail of those previously exposed, but provide an approximation to enable a greater understanding of the obtained results.

## Data Availability Statement

The original contributions presented in the study are included in the article, further inquiries can be directed to the corresponding author.

## Ethics Statement

This research has been reviewed and approved by the Research Ethics Committee of the Department of Applied Communication Studies of the Media and Communication Science School, Complutense University of Madrid. All of the participants were notified of their voluntary participation and anonymous contribution, and informed consent was given following the guidelines of the Declaration of Helsinki. The patients/participants provided their written informed consent to participate in this study.

## Author Contributions

LM-V, I-AG-V, and CL-B have carried out the literature review and data collection, have designed the research project, analyzed the data, and jointly wrote the manuscript. All authors contributed to the article and approved the submitted version.

## Conflict of Interest

The authors declare that the research was conducted in the absence of any commercial or financial relationships that could be construed as a potential conflict of interest.
